# Prevention and Recovery Care Services in Australia: Developing a State-Wide Typology of a Subacute Residential Mental Health Service Model

**DOI:** 10.3389/fpsyt.2019.00383

**Published:** 2019-06-11

**Authors:** Carol Harvey, Lisa Brophy, Holly Tibble, Helen Killaspy, Matthew J. Spittal, Bridget Hamilton, Priscilla Ennals, Richard Newton, Paul Cruickshank, Teresa Hall, Justine Fletcher

**Affiliations:** ^1^Department of Psychiatry, The University of Melbourne, Parkville, VIC, Australia; ^2^Psychosocial Research Centre, NorthWestern Mental Health, Coburg, VIC, Australia; ^3^Melbourne School of Population and Global Health, The University of Melbourne, Parkville, VIC, Australia; ^4^Mind Australia Ltd, Heidelberg, VIC, Australia; ^5^La Trobe University School of Allied Health, Human Services and Sport, Bundoora, VIC, Australia; ^6^Division of Psychiatry, University College London, London, United Kingdom; ^7^School of Nursing, Faculty of Medicine, Dentistry and Health Sciences, The University of Melbourne, Parkville, VIC, Australia; ^8^Neami National, Preston, VIC, Australia; ^9^Peninsula Mental Health Service, Frankston, VIC, Australia; ^10^Wellways, Fairfield, VIC, Australia; ^11^Nossal Institute for Global Health, and Centre for Mental Health, Melbourne School of Population and Global Health, The University of Melbourne, Parkville, VIC, Australia

**Keywords:** subacute, community-based residential environment, implementation, service typology, built environment, family inclusion

## Abstract

**Aims:** Community-based residential alternatives to hospitalization are an emerging service model. Evidence for their acceptability and effectiveness is promising but limited. Prevention and Recovery Care (PARC) services are one such residential model, offering short-term subacute treatment and care (usually between 7 and 28 days). PARC services in Victoria, Australia, are designed to support consumers with severe mental illness to either avoid a psychiatric hospital admission (step-up care) or transition from hospital back into the community (step-down care). As a precursor to a series of studies investigating the appropriateness, effectiveness and efficiency of PARC services, we aimed to investigate whether a typology of PARC services can be developed.

**Methods:** A manager or other appropriately knowledgeable staff member from each of the 19 adult PARC services included in the study completed a tool based on PARC operational guidelines (the Victorian PARC service mapping questionnaire) and a validated instrument measuring the quality of care in residential mental health settings (the Quality Indicator for Rehabilitative Care, QuIRC). Thirty (of 42) stakeholders participated in a modified Delphi study to select 23 from the available 230 variables for entry into a hierarchical cluster analysis.

**Results:** Cluster analysis produced three clusters of equal dissimilarity. At the 90% confidence level, there were four variables which were significantly different between clusters. These were the year the PARC was opened, the QuIRC Living Environment domain score, the proportion of all admissions that were a step-down admission from an inpatient unit, and how often families were invited to care meetings. Sensitivity analyses suggested the findings were robust to the method used to identify clusters.

**Conclusions:** Although PARC services were broadly similar, their identified differences suggest there is variable model implementation across Victoria sufficient to generate a PARC service typology. This typology may prove important for interpreting differences in outcomes experienced by consumers and carers using PARC services, when applied in our analyses of service effectiveness. The value of conducting service mapping and typology studies is underscored. Further research to characterize subacute residential services, including recovery-promoting features of the built environment, is warranted.

## Introduction

Community-based residential alternatives to hospitalization in psychiatric inpatient units are becoming increasingly available in health systems in many developed countries. The rationales for their development are varied and include the following: providing a less restrictive and potentially more home-like alternative to acute wards, which is also consistent with consumer preferences; a greater emphasis on providing recovery-oriented care; and an increased focus on reducing acute hospital admissions, especially in the context of the drive to reduce involuntary admissions and to reduce costs and pressure on inpatient beds (1–3).

Current research about community-based residential alternatives is limited and of insufficient quality to evaluate effectiveness (2, 4). However, preliminary findings are promising. A systematic review of community-based residential alternatives to acute admission, which included crisis houses, community mental health center beds and adult family placements, identified that they were equivalent or better than standard inpatient services with respect to symptomatic outcomes (4). Consumer satisfaction and cost effectiveness tended to also favor community-based alternatives, and no clinical outcomes were identified that were worse than for acute admissions (4). These findings are echoed elsewhere (2, 5), but existing research is limited by factors such as the paucity of long-term follow-up studies and the diverse and poorly defined community-based alternatives [e.g., Refs. (2, 4, 6, 7)]. One example of the definitional uncertainties is that crisis houses have sometimes been viewed as providing acute services to consumers (2) and at other times subacute services (4). Both acute and subacute services provide care to people who are experiencing a mental health crisis and/or a significant exacerbation of the symptoms of their mental illness. However, acute services typically provide more intensive support to those requiring immediate treatment, whereas subacute services provide less intensive services where the need is less imminent and tend to focus more on rehabilitation and/or recovery outcomes. One study did attempt to develop a typology of residential alternatives to standard acute psychiatric care in England using cluster analysis (1). That study classified community-based service types by services such as the following: clinical crisis houses; crisis team beds; nonclinical alternatives typically managed by the voluntary sector; and specialist crisis houses for specific groups, such as women.

In Victoria, Australia, a new subacute community-based publicly funded residential service model, Prevention and Recovery Care (PARC) services, has been implemented across the state since 2003. PARC services offer short-term (usually between 7 and 28 days) treatment and care in a residential setting and are designed to support consumers with severe mental illness to either avoid a psychiatric hospital admission (step-up care) or transition from hospital back into the community (step-down care). These services accept consumers who consent to PARC even if treated involuntarily under a Community Treatment Order (providing involuntary outpatient commitment). PARC services have a strong emphasis on integrating clinical and personal recovery-oriented care (8) with a commitment to greater consumer involvement and least restrictive practices and in this regard do not fit neatly into the aforementioned typology (1).

PARC services are operated as a partnership between nongovernment agencies (known as Mental Health Community Support Services, MHCSS, in Victoria) and local clinical mental health services. These partnerships are agreed upon following a tender process where the preferred MHCSS is chosen by the local clinical mental health service. The PARC services framework and operational guidelines were developed by the Victorian government Department of Health (now known as the Department of Health and Human Services, DHHS) to support service planning and delivery (9). Several uncontrolled, quasi-experimental or small mixed-methods studies of the Victorian PARC service model or equivalents in other states have suggested improved symptomatic, functional and recovery outcomes for consumers, although findings are mixed concerning reductions in psychiatric bed usage (10–15).

A clear definition of the PARC service model which describes how it has been implemented is required in order to develop a robust evidence base for these services. The existence of the Department of Health (9) operational guidelines assists with this, although nine PARC services were established prior to the release of these guidelines. It is apparent that some local adaptation of any service model may occur to support successful implementation in varied contexts (16), and anyway services may deviate from the described model (17). For example, even a well-defined service model such as Assertive Community Treatment with widely recognized model fidelity assessments [e.g., Refs. (17, 18)] has shown variable implementation within and between different settings and over time (19, 20). This may be explained by the “large array of contextual factors that influence implementation, interact with each other, and change over time” (21, page 2). Thus, implementation occurs within complex adaptive systems, such as health services (21, 22). It is likely that Victorian PARC services may vary given these diverse influences on the model.

The current study is one of a series of mixed methods studies within a larger project with the overall aim of investigating the appropriateness, effectiveness and efficiency of PARC services across the state. We have already used routinely collected secondary data to profile PARC consumers and compare them with consumers using inpatient services (23) and conducted a service mapping exercise to describe the role and function of all Victorian PARC services for adults with mental illnesses. We are concluding data collection for the following studies: a longitudinal study of recovery-oriented outcomes experienced by consumers and carers who have used PARC services and a qualitative study examining the implementation of PARC services from the perspective of consumers, carers and staff. For these latter two studies of consumer and carer outcomes and experiences, prior to conducting analyses, it is important to establish whether individual Victorian PARC services differ in significant ways from each other and, if so, whether a typology of different PARC services can be generated. If a typology can be generated, this can be tested as a potential explanatory variable within analyses for these studies. Therefore, the current study uses data from the aforementioned service mapping exercise aiming to investigate whether a typology of PARC services can be developed. The research question for the current study is as follows: do the PARC services cluster into particular groups with shared characteristics, differing from other groups? Our hypothesis was that there would be differences between PARC services and that these differences could be characterized by several distinct clusters.

## Method

Ethics approval for this project was granted from the University of Melbourne’s Human Research Ethics Committee (project number 1647880.1).

## Study Setting

The study planned to include all 19 PARC services providing subacute community-based residential services to support adults with mental ill-health in Victoria and which were operating at the time of study commencement.

## Participants

Each of the adult PARC services nominated a manager or other appropriately knowledgeable staff member to participate in a service mapping exercise (*n* = 19 staff participants). Each manager was sent a letter explaining the project and the types of information that the nominated staff member would need to know to complete the assessment tools at a forum. The nominated staff member was provided with the plain language statement and a consent form. In addition to this group of participants, a second group of 29 stakeholders (described below) participated in a modified Delphi study to select the variables that were entered into a cluster analysis. A Delphi study is a well-established method for collecting, organizing, reviewing and revising the opinions of panels of individuals who generally do not meet face to face (24, 25). The Delphi method is a systematic and iterative process that allows equal weighting of participants’ views and renders the process of determining priorities transparent (26).

## Data Sources

Two data sources were used in this study.

1. The **Victorian PARC service mapping questionnaire** was developed specifically for the service mapping exercise. It was designed to collect data concerning the adherence of each PARC service to the Department of Health’s (2010) PARC services framework and operational guidelines. Details about the types of services offered were also collected.
2. The **Quality Indicator for Rehabilitative Care (QuIRC)** is an international quality assessment tool for longer term inpatient and community-based mental health facilities with excellent interrater reliability and criterion validity (27). Although completed by the service manager, ratings correlate well with consumers’ experiences of the service, including the degree to which it promotes their autonomy (28). The QuIRC comprises 145 items that provide a combination of descriptive data and data that are collated into percentage scores on seven domains of care, with higher scores reflecting better quality on that domain. The domains are as follows, with an example item from each in brackets: Living Environment (“What do you think of the general condition of the building outside?”); Treatments and Interventions (“How many families of your current patients/residents have had family psychoeducation in the last 12 months?”); Therapeutic Environment (“How often do you have meetings where staff and patients/residents discuss the running of the facility?”); Self-Management and Autonomy (“Do your patients usually prepare their own meals (with support if necessary)?”); Social Interface (“How many of your residents have regular contact with nonservice user friends?”); Human Rights (“Is a welfare/benefits advice service available to your patients/residents?”); Recovery-Based Practice (“Do clients who have legal capacity have full control over their finances?”). However, given that PARC services aim for a short length of stay, some adaptations were made (e.g., items asking about consumers’ receipt of specific therapeutic interventions in the past 12 months were altered to receipt during the last month; the item “staff are hopeful that consumers will move on” was modified to read “staff are hopeful that consumers will make some progress on their recovery goals”).

## Data Collection

In early 2017, a forum of all nominated staff participants was convened for administering the Victorian PARC service mapping questionnaire and the QuIRC. Each staff member was provided with an iPad to complete the assessment tools, and members of the research team were available to clarify any questions that arose. Two services were unable to allocate an appropriate staff member on the day of the forum, and so were guided individually through the tools on a separate occasion by a member of the research team. To preserve confidentiality, each PARC was allocated a unique identifier so that neither the MHCSS responsible for operating the PARC nor the lead clinical mental health service could be identified in the data.

### Modified Delphi Exercise to Select Variables for Entry into the Cluster Analysis

A total of 230 variables from the Victorian PARC service mapping questionnaire and the QuIRC were available to describe each PARC. For the cluster analysis, this large set of variables had to be reduced to a smaller subset, the components of which were judged as most likely to demonstrate any variation between the 19 PARC services. To do this, we conducted a modified Delphi exercise (see below).

We added two variables we felt were potentially useful discriminatory variables for the cluster analysis and included them in the Delphi exercise. These were the following:

- *Proportion of step-down admissions in a month*: This variable was chosen because previous Australian research has identified different service goals and characteristics for step-down and step-up service functions (13, 14). The proportion of all PARC admissions that were a “step-down” admission from an inpatient unit, in 2015 and 2016, was calculated from admissions data provided by the Victorian Government DHHS. DHHS data were used since they were more likely to reflect the actual proportion of step-down admissions compared with staff participants’ estimates provided within the Victorian PARC service mapping questionnaire. An admission was considered a step-down from an inpatient unit if either a) the PARC admission “detailed description” was “Transfer from Public Mental Health Inpatient Service” or b) the time since last inpatient discharge was less than 7 days. This time frame was chosen as typical for PARC services to assess and admit consumers referred at the end of an inpatient stay.
- *The Socio-Economic Indexes for Areas (SEIFA)*: Socio-economic status was associated with mental health service use and treatment provision elsewhere [e.g., Refs. (29, 30)]. Socioeconomic data were derived from the 2011 Australian census of population and housing (31). The *Index of Relative Socio-economic Disadvantage (IRSD)* was mapped to the locality of each PARC as deciles within the state distribution for Local Government Areas. Deciles for the PARC services ranged between 2 and 10, with lower scores indicating greater social disadvantage.

There were large amounts of missing data in the step-down admissions data set for one regional PARC. Therefore, this service was excluded from all subsequent analyses, resulting in data from 18 of the 19 PARC services being subject to cluster analysis.

The process for conducting the modified Delphi exercise was as follows:

1. The seven QuIRC domain scores were retained for inclusion in the cluster analysis by default because they represented useful and internationally relevant summary variables.
2. Descriptive statistics (means, standard deviations and proportions) were derived for the 230 variables, enabling initial examination of the distributions of all variables across the PARC services. These statistics were examined by two of the coauthors (CH and JF) in order to select a “long list” of all variables (*k* = 36) that showed variation and thus had potential to distinguish differences between PARC services.
3. An on-line survey was developed for an expert group of stakeholders (*n* = 42) which comprised the following: advisory group members who had lived the experience of PARC services as consumers and/or carers; investigators and partners with service and/or academic knowledge and expertise concerning the PARC service model; research assistants involved in visiting PARC services to recruit consumer and carer participants for the longitudinal study of experiences of PARC services. Thirty of the 42 expert stakeholders participated in the on-line survey (one refused, five could not be reached for contact, and six were unavailable at the time of the exercise). The long list of promising variables (*k* = 36) was presented to each participating stakeholder with instructions to use their expertise to select a subset of about 20 variables judged to be most important in classifying PARC services into groups. The survey also allowed respondents to suggest other variables which they thought might be important in distinguishing PARC subgroups. Thirteen of the 30 respondents each suggested between one and seven additional variables or factors.
4. Stakeholder survey responses were then examined, and variables with a low rate of endorsement were dropped from the list unless there was support for their retention from existing literature. Additional suggested factors were also dropped if there was no equivalent variable in the available data set or obtainable from routine data. This resulted in an interim list of 32 variables.
5. Finally, these 32 variables were presented, with their source, rates of endorsement and any other relevant background information and discussed by a subgroup of 7 of the original 30 stakeholders (with MHCSS, clinical and academic expertise). All the variables previously removed from this list together with their reasons for removal were presented and discussed, and their removal was endorsed. Then, each of the 32 remaining variables was discussed until consensus was reached on a final list of 16 variables which, combined with the previously selected seven QuIRC domain scores, resulted in a set of 23 variables for entry into the cluster analysis (see **Table 1**, column 1, for the full list of variables included in the cluster analysis). Thus, the final selection was undertaken by the subgroup of seven stakeholders, but it was informed by the initial quantitative analysis and sorting of 230 variables followed by the responses in the on-line survey from 30 stakeholders that included consumers, carers, and staff.

**Table 1:** Characteristics of the three identified clusters of PARC services (*n* = 18).

Variable	Cluster A (N = 3)	Cluster B (N = 11)	Cluster C (N = 4)	p-value
*PARC Opening Year	2004 to 2006	2009 to 2013	2014 to 2017	<0.001
Families are invited to care meetings (1 = never, 2 = occasionally, 3 = sometimes, 4 = usually, 5 = always)	Mean 2 Range 1–3	Mean 3.9 Range 2–5	Mean 4 Range 3–5	0.026
Proportion of step-down admissions (0–1)	Mean 0.4 0.3–0.5	Mean 0.5 Range 0.3–0.7	Mean 0.3 Range 0.2–0.4	0.062
QuIRC Domain: Living Environment (%)	Mean 71.3, Range 66–78	Mean 71.5, Range 58–82	Mean 81.5, Range 76–92	0.089
QuIRC Domain: Recovery-Based Practice (%)	Mean 65.1 Range 63.4–67.5	Mean 64.2 Range 58.5–72.1	Mean 69.6 Range 63.0–76.5	0.117
*Does your PARC employ peer workers	Yes: 1 No: 2	Yes: 5 No: 6	Yes: 4 No: 0	0.132
*MHCSS to AMHS staff Full Time Equivalent (FTE) ratio	Mean 4.8, Range 4.5–5.0	Mean 3.0, Range 0.5–7.0	Mean 2.2, Range 1.4–4.5	0.259
QuIRC Domain: Treatments & Interventions (%)	Mean 68.5 Range 63.9–74.4	Mean 63.4 Range 53.0–74.0	Mean 58.3 Range 50.7–76.2	0.304
QuIRC Domain: Self-Management & Autonomy (%)	Mean 72.4 Range 68.6–75.7	Mean 71.3 Range 64.9–80.6	Mean 75.6 Range 71.1–85.0	0.323
QuIRC Domain: Therapeutic Environment (%)	Mean 60.7 Range 59.1–61.6	Mean 63.0 Range 60.0–73.3	Mean 64.8 Range 60.7–73.9	0.442
QuIRC Domain: Human Rights (%)	Mean 73.8 Range 69.4–77.7	Mean 69.4 Range 64.6–82.7	Mean 70.6 Range 59.6–80.8	0.526
*How many AMHS and MHCSS joint policies does the PARC have (maximum possible 7**)	Mean 5.0, Range 1–7	Mean 3.6, Range 0–6	Mean 4.3, Range 4–5	0.564
Staff are hopeful that consumers will make some progress on their recovery goals***(1 = almost no one, 2 = around one quarter, 3 = around one half, 4 = around three quarters, 5 = almost everyone)	Mean 4 Range 3–5	Mean 4.5 Range 3–5	Mean 4.3 Range 4–5	0.602
Staff FTE per bed	Mean 0.9 Range 0.6–1.1	Mean 1.2 Range 0.7–3.2	Mean 0.9 Range 0.8–1.0	0.635
Proportion of consumers that regularly take part in unit activities (0 to 1)	Mean 0.7 Range 0.5–0.8	Mean 0.8 Range 0.5–1	Mean 0.8 Range 0.5–1	0.649
Psychiatrist FTE per bed	Mean 0.05 Range 0.0–0.2	Mean 0.03 Range 0.0–0.1	Mean 0.05 Range 0.0–0.1	0.656
IRSD decile (1 to 10)	Mean 5.3 Range 2–10	Mean 6.6 Range 2–10	Mean 7.3 Range 5–9	0.682
Number of therapeutic modality training modules undertaken by staff (maximum possible 11****)	Mean 6.7 Range 3–11	Mean 5.3 Range 2–9	Mean 5.8 Range 3–9	0.753
Proportion of consumers that regularly take part in activities in community (0 to 1)	Mean 0.4 Range 0.0–0.8	Mean 0.4 Range 0.2–0.5	Mean 0.3 Range 0.1–0.6	0.770
QuIRC Domain: Social Interface (%)	Mean 69.6 Range 64.1–77.6	Mean 66.9 Range 45.4–78.0	Mean 69.9 Range 64.4–76.1	0.812
Decisions about care are negotiated with consumers (1 = not at all, 2 = a little, 3 = somewhat, 4 = quite a lot, 5 = a great deal)	Mean 4.3 Range 3–5	Mean 4.3 Range 3–5	Mean 4 Range 3–5	0.836
Staff would be happy to have a family member stay at the PARC (1 = not at all happy, 2 = not very happy, 3 = neither happy nor unhappy, 4 = quite happy, 5 = very happy)	Mean 4.7 Range 4–5	Mean 4.5 Range 3–5	Mean 4.5 Range 4–5	0.910
PARC Location	Inner City = 0 Suburbs = 2 Regional = 1	Inner City = 2 Suburbs = 8 Regional = 1	Inner City = 1 Suburbs = 2 Regional = 1	0.744

One of the 19 adult PARC services was excluded from the cluster analysis due to missing admissions data.

*Indicates variable from the Victorian PARC service mapping questionnaire.

**Seven policies for PARC services: Guidelines for entry and exit; procedural document for admission and discharge; risk assessment protocols; critical incidents policy; staff education and training policy; complaints policy; model of staff structure.

***QuIRC item adapted for this study.

****11 types of training enquired about in the QuIRC. Patients’ rights; mental health law; communication skills; recovery-based practice; de-escalation techniques; talking therapies (e.g., counseling, psychotherapy, Cognitive Behavioral Therapy); family work (e.g., psychoeducation); health promotion (e.g., exercise and diet); smoking cessation; alcohol and drug misuse; work skills and employment.

### Statistical Analysis

Cluster analysis was undertaken to classify the 18 PARC services included in the analysis into groups of similar (within a class) and dissimilar (between classes) services. We did this using hierarchical cluster analysis using the weighted average linkage method. All 23 items identified using the modified Delphi study were entered into the model. The resultant clustering was displayed using a dendogram. Weighted average linkage is an agglomerative method—it brings items together into a larger cluster rather than dividing them into smaller clusters. Primary distinctions between clusters were examined by regressing scores on the clusters or using chi-square tests for nonordinal categorical variables. As a sensitivity analysis, clustering was repeated using single linkage (merging clusters based on the distance between the two closest elements) and complete linkage (merging clusters based on the distance between the two furthest elements) in order to determine how influential the clustering method was on the resultant clusters.

### Results

The 18 PARC services included in this analysis opened between 2004 and 2017. The regional service which was excluded due to missing data opened in 2003. The PARC services were mostly in suburban locations within the state capital city (the PARC in the second largest Victorian city was also classified as suburban): inner city (3), suburban (13), and regional (3). Seven different MHCSS operated at least one PARC, ranging from one through six. Twelve clinical mental health services were involved in auspicing a PARC service, most only one. Allocated staff per PARC bed varied widely, from 0.6 to 3.2 full time equivalents (FTEs). The mean allocated psychiatrist (FTE) per bed was 0.04 (SD = 0.05), with six PARC services (one third) having no specifically designated psychiatric input. All the studied PARC services provided both step-up and step-down admissions, with the proportion of all admissions that were a “step-down” admission varying from 0.2 to 0.7 (mean 0.4, SD = 0.2).

**Figure 1:**
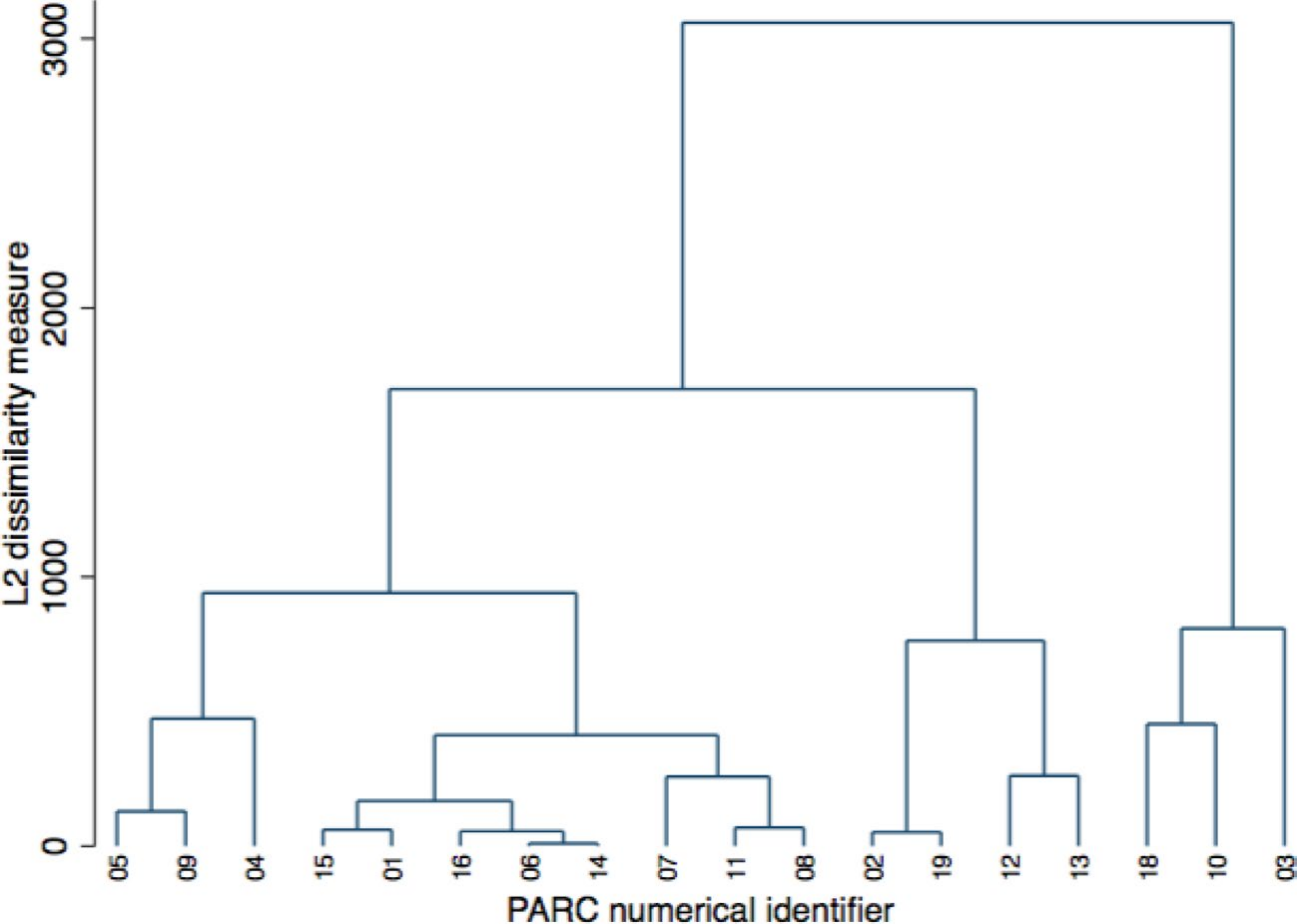
Cluster dendogram for 18 Adult PARC services using weighted-average linkage.

Following cluster analysis, three clusters of equal dissimilarity were observed within the dendogram (**Figure 1**). One cluster contained 3 PARC services (cluster A), one contained 11 services (cluster B) and one contained 4 services (cluster C). PARC services 14 and 06, which are colocated, had no discernible dissimilarity. At the 90% confidence level, there were four variables which were significantly different between clusters. These were the year the PARC was opened, the QuIRC Living Environment domain score, the proportion of all admissions that were a step-down admission from an inpatient unit, and how often families were invited to care meetings.

**Table 1** presents the characteristics of each PARC cluster, according to average scores (mean and range, numbers or date range, as appropriate). Those MHCSS which operated more than two PARC services were represented in all three clusters. Among the three largest clinical mental health services which led between two and four PARC services each, two clinical mental health services were represented across two clusters and PARC services associated with the third clinical mental health service were only found in the largest cluster (B).

The three PARC services in cluster A were founded between 2004 and 2006, making them the longest running PARC services under continuous management in Victoria. Like cluster B PARC services, they had lower scores for the QuIRC Living Environment domain compared with cluster C. Cluster A PARC services fell between the other two clusters for average proportion of step-down admissions. On average, families were invited to care meetings occasionally, which was less frequently than by PARC services within the other two clusters. The 11 services in cluster B were opened between 2009 and 2013. Like cluster A they had lower scores for the QuIRC Living Environment domain; however, families were invited to care meetings more frequently within cluster B PARC services compared with cluster A (that is, usually rather than occasionally). These were the services with the highest average proportion of step-down admissions of all three clusters. The four services in cluster C were opened during or after 2014. They had the highest scores on average for the QuIRC Living Environment domain and, like cluster B services, usually invited families to care meetings. However, PARC services within cluster C had the lowest average proportion of step-down admissions of all three clusters.

Sensitivity analysis using the complete linkage method resulted in the identical set of three clusters. Sensitivity analysis using single linkage did not identify any late joining outliers. Taken together, these additional analyses suggest that the findings are robust to the method used to identify clusters.

## Discussion

### Key Findings

As part of a service mapping exercise to develop a PARC service typology, we conducted a hierarchical cluster analysis which classified 18 of the adult PARC services in Victoria, Australia, into groups of similar and dissimilar services. Three clusters were identified, characterized by differences according to the year the PARC was opened, the QuIRC Living Environment domain score, the proportion of all admissions that were a step-down admission from an inpatient unit, and how often families were invited to care meetings. This typology will be used within our larger project investigating the effectiveness and efficiency of PARC services. The identified clusters—and the variables which characterize them—will be tested as explanatory variables for differing outcomes experienced by consumers and carers using PARC services. Two of the four discriminatory variables were derived from the Quality Indicator for Rehabilitative Care, suggesting this instrument may be useful for similar exercises.

The QuIRC Living Environment domain includes aspects of the built environment and how the facility is organized and run. These include building condition, décor, cleanliness and availability of outside space, whether the amenity is homely in appearance, whether residents can move freely in and out and around the facility, and visitor access. Our finding of significant differences between PARC services according to this domain may prove important for residents’ outcomes, since Living Environment domain scores were significantly positively associated with residents’ quality of life in European longer-term mental health facilities (28). Similarly, features of the inpatient environment are recognized as potential contributors to consumers’ recovery (32).

The PARC services were clustered according to year of opening, and one can hypothesize that the differences in the QuIRC Living Environment domain score may simply reflect an environment that has been more recently built, with the four most recently opened services benefitting most from purposeful design (33). Indeed, all four cluster C facilities fell into this category (Fletcher et al., in submission), lending support to this explanation. High scores on this domain may also reflect free movement of consumers inside and out of these environments. This may be more important for residents than, for example, the absence of restrictive practices that are common in acute wards and has been linked with differences in staff-consumer therapeutic alliance between inpatient and subacute settings (34, 35). Further exploration is needed to establish what the enabling aspects of PARC environments are. Since the Living Environment domain encompasses the practical aspects of how the facility is organized and operates, more recently opened facilities may have benefitted from accumulated experience in operating this new service model. It would therefore be useful to understand more about how cluster C PARC services differed in their setup from the other PARC services.

The findings regarding family invitations to care meetings and the proportion of step-down admissions do not lend themselves to easy explanations. Family inclusion has been a policy priority underpinned by growing recognition of family needs, as well as evidence of benefits to the consumer (36, 37). However, practice has lagged, so it can be viewed as a marker of a progressive model of care (38). Several policies setting out clear expectations about recognizing families and involving them in care planning and delivery were developed in the past decade in Australia [e.g., Ref. (39)]. Thus, PARC services which have opened since 2009 (that is, those in clusters B and C) may have adopted this practice into their routine model of care more than older services. Further, the involvement of families and carers is highlighted in the Victorian Framework for recovery-oriented practice (39), and this may have particularly influenced PARC services which emphasize recovery-oriented care. The many obstacles and challenges to family-inclusive practice and widespread culture change needed to embed this practice have been identified (15, 38), but we did not find that the local MHCSS service operator was a factor which distinguished between clusters in this regard. Our finding concerning family invitations to care meetings may also speak to increasing social openness of the PARC services. Where families are readily welcomed into an inviting physical setting and included in care planning, the model may be less institutional, more accessible to members of the community more broadly, and more transparent and accountable in its operations (40).

The proportion of step-down admissions varied significantly across PARC services, and this may have important implications for consumers’ clinical and recovery outcomes, since differences between step-up and step-down clients with respect to their clinical and functional profile and associated needs have been reported (13, 14). The proportion of step-down usage was lowest for cluster C, the most recently opened group of PARC services. A simple explanation may be that the environment of newer PARC services was more inviting to consumers and families in the community. PARC services operate as part of a complex mental health service system (22), with system components, such as inpatient services and community services, playing key roles in driving local service usage as they create pressure for accepting consumers from either setting (3, 41). Our finding suggests variation in the systems within which these PARC services operate rather than a choice made by the PARC alone. It may also be that more recently established PARC services are seen to offer a tested and valued alternative to an inpatient admission prompting higher referrals from community services. Consistent with this, community alternatives to admission were rated as providing more involvement and support by residents and staff (42, 43) and seen as more collaborative and informal by stakeholders from other parts of the service (43, 44). However, as data were gathered for this study at a single time point, it is impossible to determine how proportions of step-up or step-down admissions have varied over time for any of the PARC services or clusters. It may be that different parts of the service system vary in the time taken to adapt to a new component entering the service system, so the proportion of step-down admissions from cluster C PARC services may increase over time (45). Another potentially important issue is that funding for early PARC services was allocated in areas of lower acute bed provision, and therefore our finding may reflect variations in bed pressure across the state, although we were unable to examine this possibility.

Neither the QuIRC domain Recovery-Based Practice nor the employment of peer workers across the clusters reached statistical significance as discriminatory variables. However, these data provide some preliminary indications of greater emphasis on recovery-based practices and increasing employment of a workforce with lived experience over the study period, mirroring trends within the wider service system. Although this suggestion moves beyond the data acquired, it is also possible that, as they become explicitly more recovery focused, PARC services are moving away from a dominant clinical orientation as a subacute service. PARC services may add value to the person’s recovery through the support provided to acquire new skills not able to be learned in the ward or while in their own domestic situation (13), thereby offering a new residential recovery-oriented service option. Further investigation is needed to examine these possibilities. Although the number of staff varied widely between PARC services, these variables did not prove to be discriminatory. Another noteworthy negative finding was that social disadvantage (as measured by the IRSD) did not show statistically significant variation between the clusters, despite earlier studies which have linked social deprivation with mental health service use, including use of inpatient beds [e.g., Refs. (29, 46)]. Similarly, there was no evidence that PARC location was a discriminatory variable, despite the possibility that the larger distances between regional PARC services and consumers’ homes and communities may impact negatively on the service model. Study stakeholders also suggested that regional mental health services have fewer community-based service options for consumers. Since living in an area lacking community services has been shown to influence inpatient length of stay as well as the probability of inpatient admission (47, 48), our finding is therefore unexpected.

### Strengths and Limitations

Strengths of this study include the state-wide scope and the completeness of the data set. The use of a recognized and validated quality indicator is also a strength, although the QuIRC had to be slightly adapted for use in this setting. The findings may be limited by the recall of staff when completing the questionnaires, even though they were asked to prepare for data collection by gathering relevant background information. The variables entered in the cluster analysis, while guided by stakeholder expertise, may have excluded relevant discriminatory variables. For example, we did not include whether the facility was purpose-built or renovated which may have helped illuminate our findings. For reasons of confidentiality, we also did not include the lead clinical mental health service or the MHCSS running the PARC as cluster analysis variables, although lead agency might have helped to explain the groupings of PARC services in each cluster. Finally, our results are based on only 18 PARC services, and this may have limited our ability to detect differences between groups.

## Conclusions

Overall, the similarity between PARC services across the 23 studied variables suggests that the implementation of these services across Victoria has been largely faithful to the intended service model. Nonetheless, the identified differences between services may prove important considering positive correlations between model fidelity and outcomes have been noted elsewhere [see Ref. (17)]. Given that these services were rolled out as part of a well-described and operationalized service model within one state mental health system, our findings underscore the value of conducting service mapping and typology studies to detect variations in model implementation. We cannot say yet which PARC profile may be preferable but will investigate this by examining associations between the clusters and consumer and carer outcomes within our large longitudinal study. Further research to characterize PARC and other subacute residential services, including recovery-promoting features of the built environment, is warranted, for which the QuIRC may be considered a useful tool.

## Data Availability Statement

All datasets generated for this study are included in the manuscript and the supplementary files.

## Ethics Statement

This study was carried out in accordance with the recommendations of The University of Melbourne’s Human Research Ethics Committee with written informed consent from all subjects. All subjects gave written informed consent in accordance with the Declaration of Helsinki. The protocol was approved by the University of Melbourne’s Human Research Ethics Committee (project number: 1647880.1).

## Author Contributions

CH, JF, LB, BH, and HK designed the study, with guidance from all other coauthors. JF, CH, LB, BH, and HK collected the data, with assistance from TH and PE. CH, LB, HT, HK, MS, BH, PE, RN, PC, TH, and JF contributed to the design and conduct of the modified Delphi exercise. HT and MS analyzed the findings, with assistance from CH, JF, and LB. CH, LB, HT, HK, MS, BH, PE, RN, PC, TH, and JF contributed to interpretation of the findings and reviewed and revised drafts of the manuscript.

## Funding

The project was funded by an NHMRC partnership grant (APP1115907) and is a partnership between academic institutions, Mental Health Community Support Services (MHCSS), clinical mental health service providers and the Victorian Government.

## Conflict of Interest Statement

The authors declare that the research was conducted in the absence of any commercial or financial relationships that could be construed as a potential conflict of interest.
